# Studying dynamic sound source localization in a virtual maze with EEG and psychophysics

**DOI:** 10.3389/fnins.2026.1907950

**Published:** 2026-09-03

**Authors:** Hanna Strauss, Sabine Grimm, Sascha Feder, Jochen Miksch, Alexandra Bendixen

**Affiliations:** 1 Cognitive Systems Lab, Faculty of Natural Sciences, Institute of Physics, Chemnitz University of Technology, Chemnitz, Germany; 2 Physics of Cognition Group, Faculty of Natural Sciences, Institute of Physics, Chemnitz University of Technology, Chemnitz, Germany

**Keywords:** head movement, location deviant, oddball paradigm, predictive coding, self-generation, spatial auditory scene analysis, variable standard

## Abstract

Auditory scene analysis benefits from spatial separation of the sound sources to be disentangled. Studies characterizing the ability to localize sound sources have revealed that spatial ambiguities can be resolved by slightly moving the head. This implies that the auditory system must take into account how head movements displace the sensors (ears) relative to the sound source, in order to separate the consequences of own movement from actual movement of the sound source. Outside of the typical lab, not only the head can be turned, but also the whole body might move towards or away from the sound source. With modern virtual-reality (VR) technology including near-real-time auditory feedback, it has now become feasible to study the relations between body movement, head movement, and sound localization in a controlled manner. Here we present a VR study in which participants move through a virtual maze to locate a repetitive alarm signal. Participants’ turning decisions provide a behavioral measure of their ability to localize the sound source. In addition, we introduce occasional task-unrelated location deviations in the alarm signal to record an unobtrusive neural measure of localization ability: the Mismatch Negativity (MMN) component extracted from participants’ continuous electroencephalogram (EEG) informs us about participants’ deviance detection at the sensory level. We examine these neural and behavioral correlates of sound localization during simulated locomotion and real head movements. Our results show high behavioral accuracy and the elicitation of a robust MMN component. This indicates faithful extraction of source location although participants move relative to the sound source. Our findings are compatible with a predictive-coding framework, where the effects caused by own movement are taken into account for the interpretation of sensory signals. Yet because the range of physical variation by own movement was narrower than intended, we cannot exclude the possibility that deviance detection rested on simpler sensory mechanisms. We discuss how this constraint can be overcome in future studies to fully exploit our new paradigm for naturalistic, yet controlled studies on sound localization and auditory scene analysis under dynamic listening requirements.

## 1 Introduction

Being able to localize the origin of sounds is a fundamental function of the human auditory system. Estimating the direction and distance of sound sources helps putting the auditory information in a spatial context. This can provide useful information, for example to locate potential dangers nearby, to find the location of one’s phone that is ringing, or to decompose an auditory scene with sound sources originating from various directions (e.g., Saupe et al., 2010; Josupeit et al., 2020).

Humans are usually good at estimating the origin of sounds, especially when sound sources are located directly in front of them. In this case, the smallest spatial deviation that is still perceptible, called the minimum audible angle (MAA), can be as low as 1° (Mills, 1958; Perrott and Saberi, 1990). An increasingly lateral position of the sound source increases the MAA, as the rate at which binaural cues vary with azimuth diminishes toward the sides (Mills, 1958; Blauert, 1997). At the same time, a more lateral sound source position leads to larger physical differences of the signals reaching the two ears, making it easier to generally tell if the sound source is positioned on the left or right side (Carlini et al., 2024).

Studies measuring the MAA are usually conducted with the listener’s body and head in a fixed position and orientation. Here we investigate sound source localization under more dynamic listening requirements, when listeners are continuously moving through virtual space. One might think that body or head movements during sound presentation could interfere with the ability to localize the sound source, as its relative position to the ears changes with the movement. Yet many studies found that it is rather helpful to rotate the head for gathering additional spatial information, especially in ambiguous situations (McAnally and Martin, 2014; Carlile and Leung, 2016; Riedel et al., 2025). This implies that listeners can take their own movement into account when interpreting the signals reaching the ears—a skill that exists across sensory systems. One way to explain this skill rests on mechanisms of predictive coding and the creation of efference copies or corollary discharge in sensorimotor loops (Klier and Angelaki, 2008; Genzel et al., 2016).

To localize sounds in the horizontal plane, the human brain primarily relies on binaural cues, in particular on interaural time and level differences (Mills, 1958; Blauert, 1997). Simulating the location of sound sources via headphones by only modulating these interaural-difference cues allows listeners to make correct decisions on sound origin along the horizontal (left–right) axis, but does not lead to the natural impression of an externalized sound source (Best et al., 2020). A full simulation of sound source location requires including the head-related transfer function (HRTF) to incorporate acoustic cues for the vertical plane and source-to-listener distance (Blauert, 1997). The HRTF fully describes the sound transmission between the source and the listener’s ears, taking into account not only interaural differences but also the filtering diffraction and reflections of the sound on the body and, in particular, on the head and ear of the listener (Wightman and Kistler, 1989a; Møller et al., 1995; Palomäki et al., 2005). Faithful reproduction of source location can be achieved with individualized (Wightman and Kistler, 1989b) and general HRTFs (Wenzel et al., 1993). This is now routinely exploited for studying auditory localization with headphones.

Auditory localization abilities can be investigated with explicit behavioral measures, for example by directly asking participants about the perceived sound direction (Mills, 1958; Perrott and Saberi, 1990), or by having them point their head or hand to that direction (Recanzone et al., 1998; Lewald et al., 2000; Majdak et al., 2010). This method is popular but difficult to combine with a setting where participants are continuously moving—repeatedly asking for an explicit judgment would interfere with the naturalness of sound source localization.

Auditory perception can also be measured unobtrusively (i.e., without explicit judgments) by recording brain activity, for instance with electroencephalography (EEG). Localization ability can be captured indirectly by inserting spatially deviating sounds occasionally into a series of sounds with otherwise constant location (so-called oddball paradigm; Squires et al., 1975; Huettel and McCarthy, 2004). This paradigm exploits the fact that rare stimuli (i.e., those with unexpected sensory properties, *deviants*) receive more intense processing by the brain than frequent stimuli (*standards*). Specifically, deviants (provided their difference to the standards exceeds the resolution of the listener’s auditory system) elicit the Mismatch Negativity (MMN) component of the auditory event-related potential (ERP) (Duncan et al., 2009; Fisher and Todd, 2025; Garrido et al., 2009; Näätänen et al., 1978; Näätänen, 1992). The MMN is a negativity at fronto-central electrodes between 100 and 250 ms after the onset of the deviation, which inverts polarity at the mastoid electrodes with nose reference (Kujala et al., 2007). From MMN elicitation, one can implicitly infer that the localization accuracy of the listener’s auditory system was sufficient to detect the angular difference between the (expected) standard and the (actual) deviant location.

The MMN was originally assumed to reflect a mismatch of a newly perceived stimulus with the sensory memory trace of physically identical standard stimuli (Näätänen et al., 1978; Näätänen, 1992). Later studies have shown that the MMN can also be observed when using physically varying standard stimuli whose variation follows a certain regularity—for instance, the physical feature may continuously increase or continuously decrease (Saarinen et al., 1992; Tervaniemi et al., 1994), or alternate between two levels (Horváth et al., 2001). This has been described in the framework of predictive coding (Winkler et al., 1996): The brain creates a model of the sensory environment and predicts the next stimulus based on previous sensory input. If the actual input does not match the prediction, the MMN is elicited as a correlate of a process that signals the prediction error and the need for updating the predictive model (Garrido et al., 2009). An alternative account posits that differential adaptation underlies the generation of MMN responses by deviant sounds (May and Tiitinen, 2010; May, 2021). This account does not require a predictive mental model, but it still requires some form of memory in the neuronal representation of the sounds encountered in the imminent past. Measuring MMN provides a way to tap into the characteristics of these representations, including their time constants and the sensory information they are based on.

The MMN responds to deviations in different physical features, such as frequency, intensity, or duration of the tones (Näätänen et al., 1978; Sams et al., 1985; Paavilainen et al., 1991; Roeber et al., 2003). Crucially for the present work, the MMN is also elicited by differences in sound source location (Paavilainen et al., 1989; Schröger and Wolff, 1996; Roeber et al., 2003), and MMN amplitude increases with the amount of spatial deviation (Deouell et al., 2006; Paavilainen et al., 1989). This makes the MMN a useful indicator for studying spatial auditory scene analysis.

Even when listeners move their heads during sound presentation, deviations in sound source location still elicit the MMN. Schechtman et al. (2012) found that this holds for head-centered and world-centered deviations. Participants were looking at a speaker in front of them while standard sounds were played from a speaker 30° to their right. After visually cueing the participants to rotate their head towards a speaker 30° to their left, the next sound was either still played from the speaker on the right (head-centered deviation) or from the speaker in the center (world-centered deviation). Both kinds of deviation lead to MMN elicitation (Schechtman et al., 2012).

In reality the situation is often even more complex, as humans are usually not just turning their heads but are also moving through space relative to the sound source, which changes the location of the sound source relative to the listener as well. The challenge then is to correctly separate changes in perceived location caused by own head movements (i.e., changing the position of the ears relative to the sound source), by own body movements (i.e., changing the distance of the head from the sound source), and by changes in the actual position of the sound source in the external world.

Virtual reality (VR) can provide an environment to study the relations between (simulated) locomotion, real head movements, and sound source localization in much more realistic settings than in classical lab studies, while retaining full control over the experimental setup. Stodt et al. (2025) tested whether it is generally possible to find an MMN for virtual sound sources—initially without incorporating head or body movements. They investigated sound localization in VR compared to the real world by either playing sounds from a speaker in front of the participants (real world) or by them seeing a replica of the experimental room through a VR headset and listening to virtual sounds, simulated to originate from the speakers’ positions, through headphones. Occasionally, standard sounds were replaced by deviants coming from a different position. Results showed that participants are as good at detecting spatial deviations of sound sources in VR as they are in the real world. Moreover, MMN was elicited by the deviations, though with a slightly lower amplitude and longer latency in comparison to measurements outside of VR (Stodt et al., 2025). These results were obtained while a chin rest ensured that the head remains still.

It is now feasible to incorporate head and body movements into sound replay in VR, thanks to the availability of an open-source toolbox for near-real-time rendering of virtual acoustic scenes [TASCAR by Grimm et al. (2015, 2018, 2019); see https://www.tascar.org]^1^. TASCAR has been used for a range of research questions, also including EEG measurements—specifically for cortical speech tracking (Daeglau et al., 2025). Here, we exploit TASCAR for investigating dynamic auditory localization abilities on a sensory level, using an oddball approach with the MMN as an unobtrusive measure of sound source localization during own movement. Our aim is to establish a new paradigm that embeds the oddball sequence organically into a virtual environment, to widen the possibilities of studying natural auditory scene perception.

Auditory oddball sequences have been embedded into VR environments before, for instance to measure spare capacity of the listener (Kober and Neuper, 2012). When used with this purpose, the oddball sequences are often artificially added to the VR, with no inherent plausibility within the virtual setting. This can be puzzling or irritating for participants, and may lead them to pay different amounts of attention to the oddball sequences depending on how much they are used to this study design. Differences in attentional allocation can confound ERP measurements (SanMiguel et al., 2008; Saupe et al., 2013). Here, we develop a VR-EEG approach in which the repeating standard sequence is plausibly embedded into the virtual environment: There is a repetitive alarm signal which participants are supposed to find and switch off. To this end, participants move through a virtual maze. Occasionally, the alarm signal has a deviating position for just one presentation. We ask whether these deviants elicit the MMN although there is no physically constant location of the standard alarm signal relative to the listener. If the listener’s auditory system can take into account how their own movement changes the relative location of the sound source, MMN should be elicited by deviant sounds. Similar to earlier studies (Paavilainen et al., 1989; Deouell et al., 2006), we expect higher MMN amplitudes for larger deviation angles. Finding an MMN in such a setup would provide a proof-of-concept and foundation for further studies on sound localization under dynamic listening requirements. We decided to use comparably large spatial deviations (well beyond the MAA) in this first systematic test of our framework.

## 2 Materials and methods

### 2.1 Participants

The prerequisites for participation in the experiment were an age between 18 and 40 years and self-reported normal hearing. We did not perform any audiometric screening, with the rationale that all stimuli were supra-threshold, and that the experimental task itself would provide a measure of accuracy for participants’ spatial auditory processing. In total, 25 participants volunteered. One participant had to abort the experiment after the first two maze walkthroughs due to motion sickness; their data were excluded from further analysis. The other 24 participants consisted of 12 women, eleven men and one non-binary person. 20 participants were right-handed, three were left-handed, and one participant was ambidextrous. The participants’ age ranged from 19 to 37 years with a median age of 22 years. All participants were recruited from among students at Chemnitz University of Technology and other interested individuals via a mailing list. They were compensated either at 10 €/h or with course credit. In accordance with the Declaration of Helsinki, participants gave their voluntary informed consent prior to the beginning of the measurements. The study procedures were approved by the ethics committee of Chemnitz University of Technology (case no. 101607178).

### 2.2 Experimental stimuli and apparatus

#### 2.2.1 Virtual maze

The experiment was programmed and presented using Unity 2019.4.32f1 and Visual Studio 2019 on a Vive Pro Eye (HTC Corporation) head-mounted display. Though technically possible with this system, no eye-tracking data were recorded due to the already complex setup, and in order not to prolong the experimental session by repeated calibration and validation.

**Figure 1:**
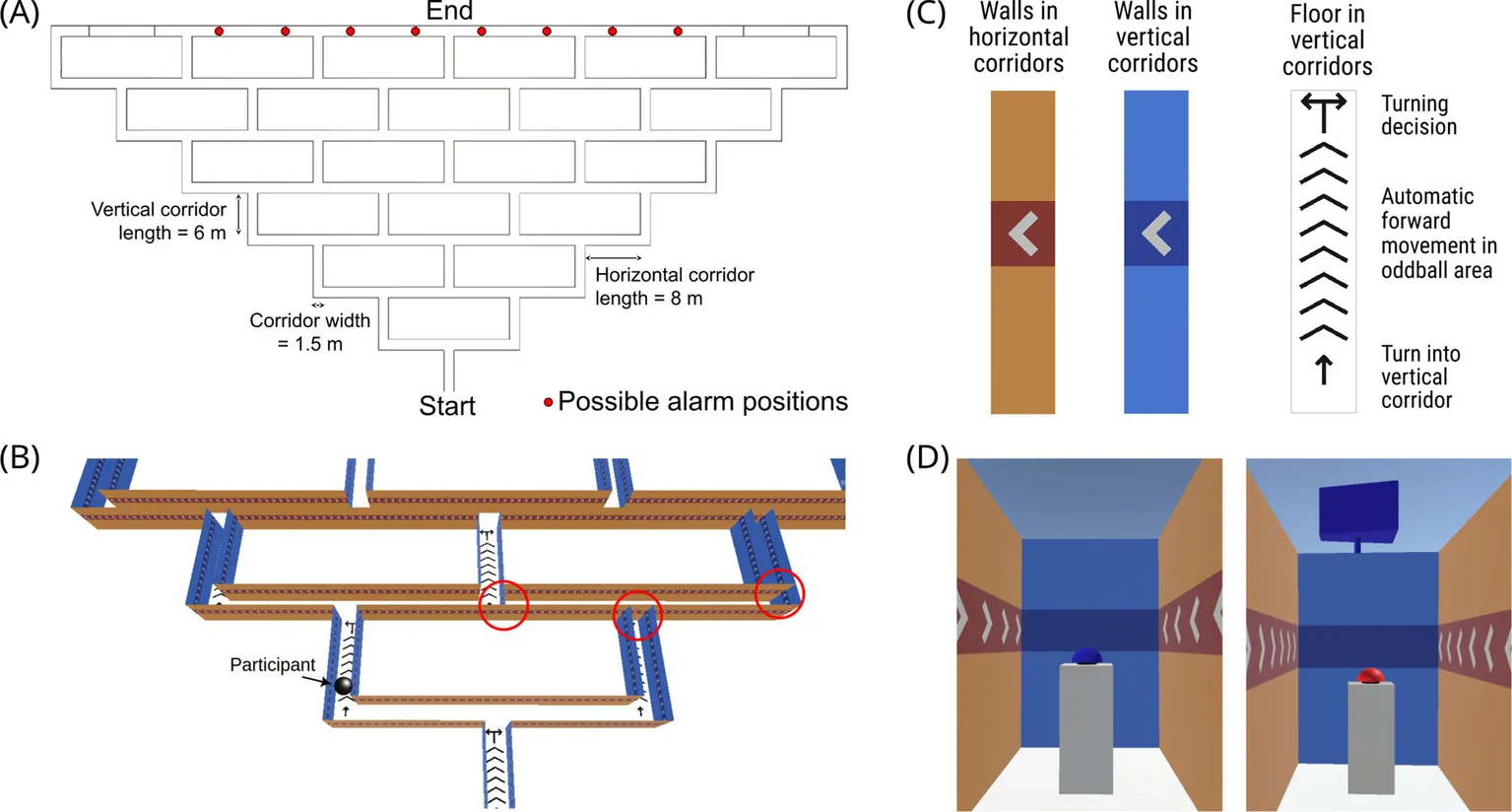
Virtual maze. **(A)** Structure and dimensions of the virtual maze used in the experiment. Labels indicate the participant’s starting position (Start) as well as the horizontal corridor to be reached at the end of a walkthrough (End). From eight predefined alarm positions (red dots), one position was randomly drawn for each walkthrough. **(B)** Example of partition walls (red circles), which were dynamically inserted into the maze well before the participant entered the respective corridor to guide them in forward direction and to the next vertical corridor nearest to the alarm. **(C)** Textures on walls and floors, which served as navigational aids. **(D)** Button objects that participants encountered in the last horizontal corridor. In the rare case of failure to find the alarm, a blue button was encountered (left image), which—when pressed—teleported the participant to the actual alarm position. In case of success to find the alarm, a red button was encountered (right image), which could be pressed to turn the alarm off. Only from this position, the loudspeaker object is visible on top of the wall (right image).

Participants moved through a virtual maze arranged in a grid-like structure (Figure 1). There are two types of corridors, which we denote *vertical* and *horizontal* in accordance with the top view of the maze shown in Figure 1A. An alarm was placed in the last (seventh) horizontal corridor at one of eight possible positions. The participant’s task was to find their way through the maze from the starting position (first vertical corridor) to the alarm, by using auditory information alone. At the end of each vertical corridor, the participant had to decide whether to turn left or right depending on the perceived direction of the alarm tone. Making correct choices at each of these turns lead to an optimal (i.e., maximally short) path through the maze, though there could be a few equally short paths in case of small turning errors. No time pressure was imposed on participants.

The maze’s configuration was dynamically updated (i.e., corridors were opened and closed) to constrain participants’ paths towards decision points that would maximize information gain for the purpose of the experiment. Specifically, in horizontal corridors, participants could only enter vertical corridors leading towards the sound source (to prevent circular paths), and only those that were as close to the alarm as possible given their previous turning decision (to prevent veering too far away from the sound source). This was achieved by inserting partition walls into the next horizontal corridor depending on the participant’s current position. Those partition walls could (i) form a corner to steer the participant to the vertical corridor closest to the alarm (see Figure 1B, left circle); (ii) conceal vertical corridors that would lead backwards (see Figure 1B, central circle); or (iii) conceal other vertical corridors that were not the one closest to the alarm (Figure 1B, right circle). Importantly, this dynamic reconfiguration happened outside the participants’ field of view, such that they experienced a stable and fully natural impression of the maze, while it ensured that they quickly returned to the optimal path even after erroneous decisions.

All corridors had a width of 1.5 m, each vertical corridor was 6 m long, and the length of horizontal corridor segments (from one corner to the next) was 8 m (see Figure 1A). To help participants navigate through the maze, walls in the vertical corridors were blue and walls in the horizontal corridors were orange. At mid-height (around 1.35 m above the ground) there were arrows on the walls pointing in the current movement direction. At the end of each horizontal corridor, an arrow on the floor marked the entrance to the vertical corridor to be taken next. At the end of each vertical corridor, arrows pointing left and right indicated that participants had to take a left/right decision. The textures used on the walls and the floor of the maze (Figure 1C) were modeled using Blender 4.1.

#### 2.2.2 Alarm

The alarm track consisted of repeating 1,000-Hz sine tones with a duration of 100 ms (including rise and fall times of 5 ms each) and an inter-stimulus interval of 500 ms, in line with recommendations for MMN recording (Duncan et al., 2009). The modeled physical intensity of the alarm at source remained constant. The intensity at the participant’s ears (played through headphones) depended on the participant’s position in the maze and thus on the distance between participant and alarm, ranging from 45 dB(A) to 72 dB(A).

Tones were presented via Sennheiser HD 25–1 II headphones. They were played back using the Toolbox for Acoustic Scene Creation and Rendering (TASCAR; Grimm et al., 2015, 2018, 2019) and transmitted via the JACK Audio Connection Kit.^2^ With each new video frame, TASCAR received information from Unity regarding the position and orientation of the VR headset and the simulated alarm tone’s location. This information was transmitted via UniOSC^3^ using the Open Sound Control protocol via a LAN connection. Based on the relative position of alarm and participant as well as the participant’s head orientation, TASCAR realistically simulated the auditory environment in the maze and modulated the tones as if they originated at the position of the alarm. This was done in near-real-time using HRTF calculations based on the Spherical Head Model (Brown and Duda, 1998). The simulation allowed participants to experience realistic auditory consequences of their locomotion through the VR and of their head turns. To ensure precise and consistent timing of tone playback, TASCAR ran separately from Unity on an extra laptop operating under a Linux environment (Ubuntu).

The virtual sound source was positioned in the last horizontal corridor of the maze, always indented half a horizontal corridor length from the closest vertical corridor (Figure 1A). Vertical partition walls were inserted at all possible alarm locations to create dead ends at the end of the maze, and the alarm was placed on top of one of those partition walls (at the height of 2.8 m). To avoid monotonous turning behavior, strongly lateralized alarm positions were not used, leaving a total of eight possible alarm positions (Figure 1A). Each position occurred in exactly three of overall 24 maze walkthroughs for each participant. The order of alarm positions was randomized separately for each participant with the restriction that no alarm position occurred more than twice in a row.

The alarm itself was represented by a loudspeaker object modeled in Blender 4.1 (Figure 1D, right). The walls in the maze were high enough to conceal the speaker up until the participant entered the last horizontal corridor, ensuring that participants could only rely on auditory cues to find the alarm’s location. To turn the alarm off, participants encountered a red button on a pillar standing on the floor 1 m in front of the alarm’s position (Figure 1D, right). By triggering that button with the VR controller, participants could stop the alarm and end the walkthrough. In case they did not find the alarm and arrived at a dead end, participants encountered a blue button (Figure 1D, left). Using that button teleported participants in front of the actual alarm, enabling them to move forward another meter and then trigger the red button to stop the alarm.

**Figure 2:**
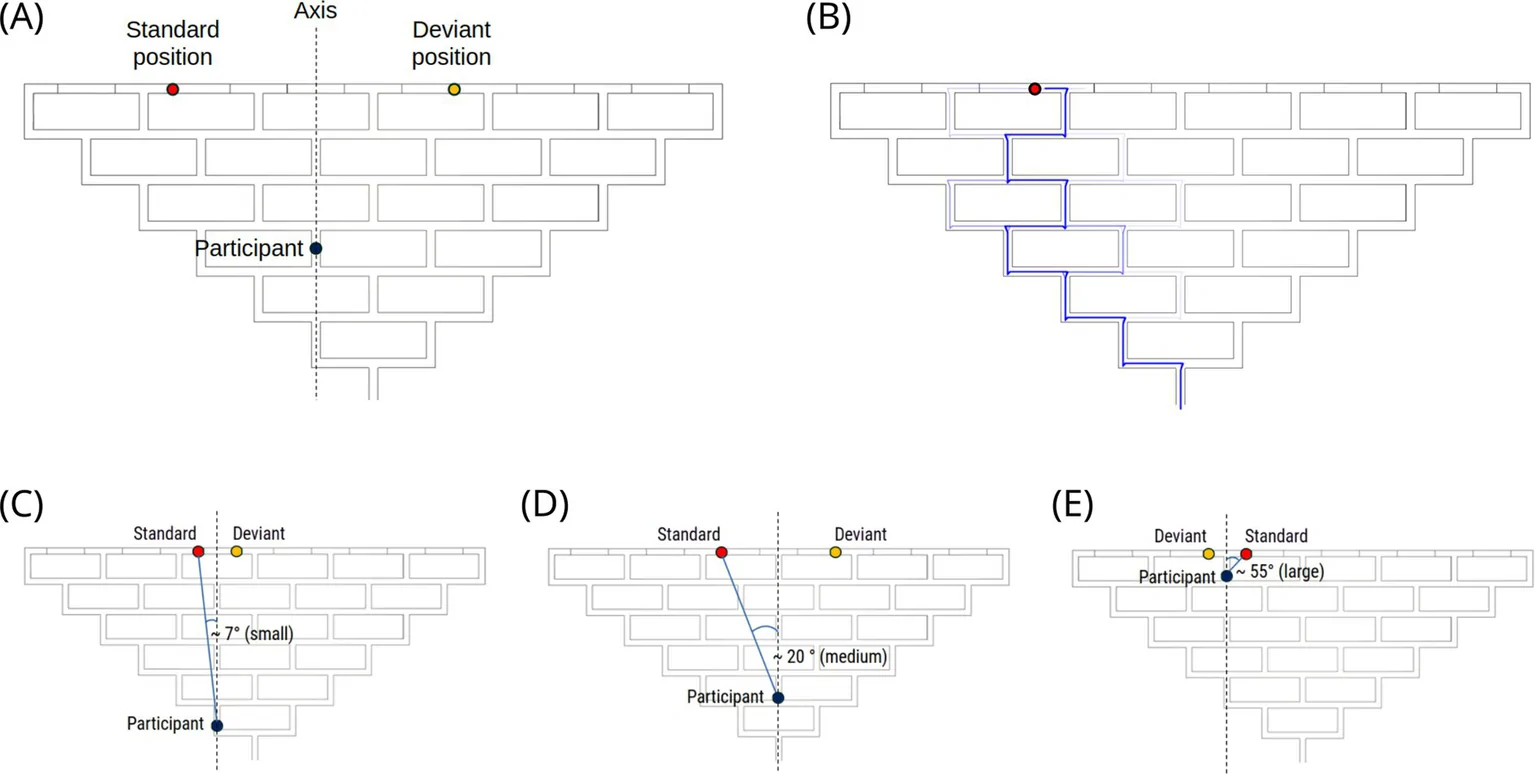
**(A)** Schematic illustration of an example alarm position during a deviant trial (yellow dot). Based on the standard alarm position of the current walkthrough (red dot) and the participant’s current position in the maze (black dot), the deviant occurred mirror-symmetrical relative to the current walking direction and thus always in the other hemifield. **(B)** Participants’ empirically observed trajectories (blue lines) for one example alarm position (red dot). The thickness of the blue lines reflects the number of trials across all participants for which the respective path was chosen. In the majority of cases, participants chose the optimal trajectory to the alarm sound. **(C–E)** Illustration of exemplary participant positions (black dot) and alarm sound positions in a standard (red dot) or a deviant (yellow dot) trial that resulted in either a small **(C)**, medium **(D)**, or large **(E)** angle size.

Deviant tones only occurred in vertical corridors. Each vertical corridor contained an *oddball area*. The size of this area was such that exactly ten tones were presented while participants passed through it. One of them (the deviant) differed from all other tones in terms of the simulated alarm’s source location (Figure 2). Note that all ten tones slightly differed due to the participant’s movement towards the source and their head movements, but the deviant tone additionally differed in the source’s location. The deviant location was a horizontal displacement symmetrically mirrored relative to the corridor’s midline, which was also the participant’s direction of movement (Figure 2A). The deviant randomly occurred in position 4, 5, 6 or 7 of the 10 tones occurring in the oddball area. Keeping the first three tones as standards ensured a stable predictive model of the sound source (Bendixen et al., 2007). Having the last three tones as standards was chosen in order not to confuse participants in their left–right decision at the end of the vertical corridor.

Figure 2 illustrates how the participant position in the maze and the virtual location of standard and deviant tones affected the angle between the participant’s movement direction and the tones. Due to the configuration of the maze and the high homogeneity of the paths taken (Figure 2B), certain ranges of angles had fewer observations than others. For later analysis, the observed angles were grouped into three equally populated categories (small, medium, and large angles; for illustration, see Figures 2C–E). This balances signal-to-noise ratio and also facilitates comparison to earlier location MMN studies, which often treat spatial separation as a categorical factor (Paavilainen et al., 1989; Deouell et al., 2006; Bennemann et al., 2013; Freigang et al., 2014).

#### 2.2.3 Movement and turning decisions

Participants’ movements through the virtual maze were a mixture of self-initiated and automatic forward movements. In horizontal corridors, participants moved by clicking on the trackpad of the HTC Vive Controller they were holding in one of their hands; they could stop clicking (and thus stop moving) at any point in time. In vertical corridors, participants were moved forward automatically to keep them from stopping while moving through oddball areas. Movement speed (whether self-initiated or automatic) was always at 1 m/s. Though possible by design, participants hardly ever stopped in horizontal corridors (on average 3.25 times in 168 horizontal corridors); hence the actual movements were similar in all corridors.

At the end of each corridor—that is, whenever participants reached a crossing or a corner and could not keep moving forward—movement through the VR was automatically stopped. Participants then clicked on the left or right side of the controller’s trackpad to turn towards the respective side. While participants were virtually rotated and placed at the beginning of the next corridor, the VR screen turned black for 100 ms to minimize any risk of motion sickness.

As horizontal corridors always ended in corners, there was only ever one turning direction to reach the next corridor. Participants remained in place until they clicked the correct side of the controller trackpad. At the end of vertical corridors, participants had to make a choice between turning left or right. These turning decisions are classified as correct or incorrect depending on whether the sound source was actually left or right relative to the corridor’s midline. Since the alarm was never positioned directly in front of a vertical corridor, there was always an objectively correct option.

Turns and teleports never happened while a tone was played. A sudden change in the simulated position of the participant would cause the tone to be instantaneously played from a different origin, which could lead to acoustic artifacts (distortions) and would in any case sound unnatural. To avoid this, participant-initiated turns and teleports were delayed until after the current tone presentation. Given the brief tone duration (100 ms), this delay was hardly noticeable by participants (Attig et al., 2017; Feder et al., 2023), thus not reducing the naturalness of the VR. Delaying turns and teleports rather than tone presentations was important to keep the inter-stimulus interval constant, in line with a repetitive alarm tone. In order to ensure that turns and teleports are not executed during tone presentation, the Unity VR software needed information about tone timing. For this purpose, the audio signal from TASCAR was also transmitted to the computer running the VR simulation (Unity), which then inhibited turns and teleports whenever a tone was present until the tone had ended.

#### 2.2.4 EEG recording

Participants’ brain activity was measured using the stationary actiCHamp EEG system with 32 active Ag-AgCl electrodes (ActiCap Slim). 29 of these electrodes were placed according to the international 10–20 system (Chatrian et al., 1985) via an elastic cap on the participant’s scalp (at the positions Fp1, Fp2, F7, F3, F1, Fz, F2, F4, F8, FC5, FC1, FCz, FC2, FC6, T7, C3, C1, Cz, C2, C4, T8, CP5, CP1, CPz, CP2, CP6, P7, Pz and P8). One electrode at the tip of the nose was used as reference electrode, and the remaining two electrodes were placed on the mastoids.

Electrodes that usually measure eye movements (IO1, IO2, LO1, LO2) were not used as the VR headset would have interfered with the precise positioning of the electrodes and potentially created artifacts by putting pressure on them. The occipital electrodes O1 and O2 were not used to accommodate the back-strap of the VR headset. Instead, sampling was denser in the frontrocentral area based on the expected topography of the MMN component (Kujala et al., 2007). The electrode choice was based on an earlier EEG-VR study using a similar setup (Feder et al., 2023). The EEG signals were recorded with a sampling rate of 500 Hz. Impedances on all electrodes were less than 20 kΩ after preparing the EEG on each participant.

In order to record the timing of the tone onsets as triggers in the EEG data, the audio signal was also forwarded to an A/D converter (StimTrak, Brain Products), and the digital (tone on/off) signal was passed to the trigger input of the EEG amplifier. Further, it was necessary for the computer running Unity to signal that a tone was a deviant to the EEG system, while keeping these systems electrically (galvanically) decoupled. For this purpose, we coupled the systems optically. Specifically, a deviant placement in Unity was accompanied by lighting an LED on the same computer, which was measured by a photodiode that connected to the digital trigger input of the EEG amplifier via a custom-programmed microcontroller (Arduino).

Whenever the VR was running, Unity recorded the following parameters with every frame: the current system time, position and orientation of the VR headset, the alarm position, whether the participant was in an oddball area, whether their last turn was correct, and their distance and angle to the alarm. This information was later used for EEG and behavioral data analysis.

### 2.3 Procedure

The experiment started for each participant with a short demographic questionnaire that asked for age, gender (optional), handedness, their abilities to see and hear, as well as their current levels of concentration and tiredness. Participants were then instructed on how to move through the virtual maze, and were given the task to search for the alarm and turn it off by using the red button at the end of the maze. They were placed in an electrically shielded and acoustically attenuated room, where they sat in a revolving chair and started with two short VR walkthroughs for training in a smaller version of the maze with only four horizontal and vertical corridors each (i.e., four oddball areas). During these walkthroughs, the participants were encouraged to ask questions if they were uncertain about the procedure. In addition to practicing the task, the purpose of this training also was to check for signs of motion sickness prior to EEG preparation.

The main experiment began after EEG preparation. Participants were allowed to rotate their heads while searching for the alarm, but were also instructed to stay calm and relaxed in the chair to avoid additional artifacts in the EEG data. The main experiment consisted of 24 walkthroughs with seven oddball areas per walkthrough (Figure 1A). Depending on how often participants turned in the correct direction and how long they needed to make these decisions, one walkthrough took between 2 and 3 min, with the majority being on the lower side of that range. The average net duration of the experimental task (24 walkthroughs) was 53 min. After each walkthrough, participants had the chance to take a break. Halfway through the experiment (i.e., after twelve walkthroughs), a longer break was enforced in order to counteract potential fatigue. After the last walkthrough, the electrodes were removed and participants could wash their hair. The total duration of the session was about 2.5 h; beyond the 53 min time-on-task, this included EEG preparation and removal, VR practice, and breaks.

### 2.4 Data analysis

All data analyses were performed using Matlab R2024a and EEGLAB v2025.0.0.

#### 2.4.1 EEG data pre-processing

The EEG data for each tone presentation were first complemented by the VR data recorded in Unity regarding the participant’s current distance and angle to the alarm as well as whether they were in an oddball area. The EEG data were then cleaned from stereotypical artifacts by performing an Independent Component Analysis (ICA). Prior to ICA decomposition, a copy of the raw EEG data was high-pass filtered using a Kaiser-windowed sinc finite impulse response (FIR) filter with a 1 Hz cutoff (Kaiser *β* = 5.65326, filter order: 9056) to facilitate the reliable extraction of independent components (Klug and Gramann, 2020). The original data set was high-pass filtered using a Kaiser-windowed sinc FIR filter with a 0.1 Hz cutoff (Kaiser β = 5.65326, filter order: 9056). Both the copy and the original data were low-pass filtered using a Kaiser-windowed sinc FIR filter with a 45 Hz cutoff (Kaiser β = 5.65326, filter order: 184). Afterwards, the data were segmented into epochs ranging from −100 ms to 500 ms relative to tone onset. Epochs with amplitude changes exceeding 750 μV, representing gross and often non-stereotypical artifacts, were removed from both data sets. The remaining epochs of the copied data set were then used for the ICA decomposition, and the extracted ICA weights were transferred to the original data set. All ICs that were more than 80% likely to be attributable to muscle activity or eye movements were marked using ICLabel (Pion-Tonachini et al., 2019) and, after visually inspecting them, removed from the data. We note that ICA was able to decompose eye-movement-related components despite the lack of EEG recordings from underneath (IO1, IO2) and lateral to the eyes (LO1, LO2). To remove artifacts remaining after ICA, all epochs with amplitude changes exceeding 150 μV were excluded from further analysis.

Artifact-free epochs were baseline-corrected by subtracting the mean activity in the interval from −100 ms to 0 ms relative to tone onset. The data for each participant was then separated by stimulus type (deviant or standard) and by the participant’s current angle to the alarm. For that purpose, the angle between the participant’s movement direction and the vector from the participant to the alarm position was extracted, and the epochs were sorted into three size categories (small, medium, and large angles). The cutoff angles for these categories were determined for each participant by sorting all deviant epochs by angle size and splitting them into three bins with equal epoch numbers. On average, the cutoff angle was 11.32° (*SD* = 1.45°) between small and medium angles, and 23.16° (*SD* = 0.35°) between medium and large angles. Note that for deviant tones, the alarm position was mirrored at the axis in movement direction, thus the actual spatial displacement of the deviant tone was twice that angle size (Figure 2). The standard epochs were also sorted into three size categories using each participant’s cutoff angles for the deviant epochs. Only standard tones presented in the oddball area were used for the analysis; additionally, each standard directly following a deviant was removed from further analysis to avoid after-effects of the preceding deviant (Sams et al., 1984).

For the final analysis, on average about 54 (at least 43) deviant epochs and 437 (at least 319) standard epochs remained per participant and angle size.

Grand-average ERPs were formed from the single-participant average ERP in each condition. To examine the processing of the location deviations, difference waves were formed by subtracting standard ERPs from deviant ERPs—once across all angles together and once separately for each angle size (small, medium, large).

#### 2.4.2 Statistical analysis

Statistical ERP analysis was calculated at the FCz electrode, where the MMN component is expected to peak (Kujala et al., 2007; Jiao et al., 2021). First, sample-by-sample running *t*-tests of the difference wave against zero with FDR correction of the alpha level (Benjamini and Hochberg, 1995) were carried out to verify differential processing of standards and deviants without visual-inspection- or effect-driven analyses, to avoid circularity (“double dipping”; Kriegeskorte et al., 2009; Luck and Gaspelin, 2017). This FDR-corrected running *t*-test analysis showed a significant negativity over a long time period. Next, to determine the time interval used for statistical and topographic MMN analysis, the negative peak latency of the difference wave across all angles at the FCz electrode was used; this choice remains neutral with respect to angle size. MMN peak latency was identified at 128 ms after stimulus onset. A 50-ms wide window was symmetrically placed around this peak latency, resulting in a time interval ranging from 104 ms to 154 ms after stimulus onset for analysis. Amplitudes were then investigated with a repeated-measures analysis of variance (rmANOVA) with the factors stimulus type (deviant, standard) and angle size (small, medium, large). Note that the main effect of the stimulus type factor at FCz is for verification purposes only, as the sample-based running *t*-test already indicates that a significant standard-deviant difference was elicited for every sample in that time-range. Polarity inversion, a defining characteristic of the MMN component (Kujala et al., 2007), was examined at the mastoid electrodes (average of M1 and M2).

The correctness of participants’ behavioral turning decisions was analyzed as a function of angle size. This was done by sorting all decisions into three size categories (small/medium/large angle), akin to the classification used for EEG analysis but with the angles at the decision point (i.e., the end of each vertical corridor) as a basis for building bins with equal numbers. On average, the cutoff angle was 13.32° (*SD* = 2.53°) between small and medium angles, and 30.07° (*SD* < 0.01°) between medium and large angles. The proportion of decision errors (i.e., incorrect turns) was assessed in an rmANOVA with angle size as a three-level factor. An rmANOVA with the three-level factor angle size was also conducted on response times for turning decisions. Response times were measured as the average time per participant between the automatic stopping of the movement at the end of a vertical corridor and the click on the controller’s trackpad.

Next, we assessed whether there was a correlation of individual MMN amplitude at FCz and the proportion of errors across all angles. As the MMN is a component with negative polarity, a positive correlation would indicate that participants with stronger responses to spatial location deviants at the sensory level (numerically lower = more negative MMN values) make more accurate behavioral decisions with respect to alarm location (lower error rates). Finally, head orientation data were inspected to assess to what extent the participants’ movement of body and head impacted the physical difference between consecutive standard sounds in oddball areas, and how this relates to the angular difference between standards and deviants.

The alpha level for all statistical tests was set to 0.05. Significant ANOVA effects involving factors of more than two levels were followed up by two-tailed, paired-sample *t*-tests, where the alpha level was Bonferroni-corrected to account for multiple comparisons.

## 3 Results

### 3.1 MMN

**Figure 3:**
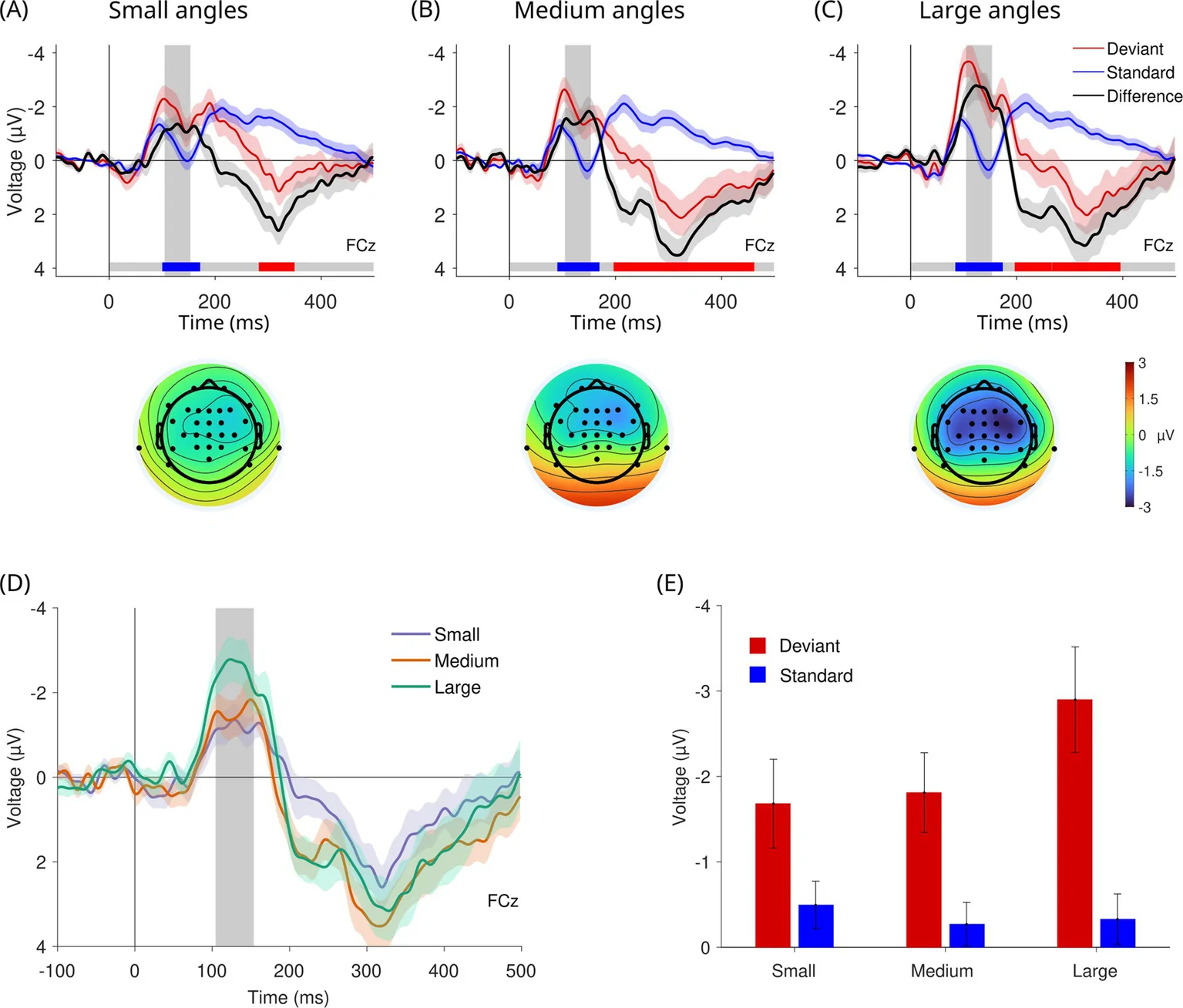
Grand-average ERPs for small, medium, and large angles. **(A–C)** Grand-average ERPs at electrode FCz elicited by alarm sounds originating from the standard positions (blue line) and from the deviant positions (red line), as well as deviant-minus-standard difference waves (black line), shown separately for small **(A)**, medium **(B)**, and large angles **(C)**. Shaded areas represent the standard error of the mean (SEM). Time windows with significant deviant-minus-standard differences according to the FDR-corrected running *t*-tests are marked in blue (in case of a negative difference) or red (in case of a positive difference) in the horizontal bar depicted at the bottom of the graph. The MMN time window is highlighted by a grey vertical rectangle. Below each graph, a 2D topographical plot of the mean amplitudes in the MMN time window is shown. **(D)** Comparison of grand-average difference waves depending on the size of the spatial deviation. Shaded areas represent SEM. **(E)** Bar graph of mean amplitudes at electrode FCz in the MMN time window depending on stimulus type and angle size, with error bars denoting SEM.

The grand-average difference waves show a significant negativity at the FCz electrode in the MMN time range for each angle size (Figures 3A–C), with numerically higher amplitudes for large deviation angles (Figure 3D). The rmANOVA shows a significant main effect of stimulus type (*F*(1, 23) = 32.47, *p* < 0.001, η_p_^2^ = 0.59), confirming MMN elicitation by deviants relative to standards. While no significant main effect of angle size was observed (*F*(2, 46) = 2.29, *p* = 0.113, η_p_^2^ = 0.09), there is a significant interaction between stimulus type and angle size (*F*(2, 46) = 3.59, *p* = 0.036, η_p_^2^ = 0.14), implying that the deviant-standard difference depends on the size of the current angle to the alarm.

Follow up *t*-tests comparing deviant and standard amplitudes (Figure 3E) show that the stimulus type effect is significant for small (*t*(23) = −3.41, *p* = 0.002, *d* = −0.70), medium (*t*(23) = −3.47, *p* = 0.002, *d* = −0.71), and large angles (*t*(23) = −5.06, *p* < 0.001, *d* = −1.03). The significant interaction of stimulus type and angle size thus indicates differences in the size of the stimulus type effect (i.e., MMN amplitude) across angles. However, none of the follow-up pair-wise MMN amplitude comparisons are statistically significant: *t*(23) = 0.68, *p* = 0.505, *d* = 0.14 for small vs. medium angles, *t*(23) = 1.90, *p* = 0.070, *d* = 0.39 for medium vs. large angles, and *t*(23) = 2.54, *p* = 0.018, *d* = 0.52 for small vs. large angles, which just fails to reach significance against a Bonferroni-corrected alpha level of 0.0167.

The topographic plots in the MMN time range (Figures 3A–C) show a pronounced frontocentral negativity for all angle conditions, with two separate negative poles visible for large angles (Deouell, 2007). Polarity inversion is visible at the mastoid electrodes and was verified by an ANOVA of the same structure as for MMN at FCz. There was a main effect of Stimulus Type (*F*(1, 23) = 10.92, *p* = 0.003, η_p_^2^ = 0.32) indicating polarity inversion, but neither a main effect of angle size (*F*(2, 46) = 0.35, *p* = 0.705, η_p_^2^ = 0.02) nor an interaction (*F*(2, 46) = 0.33, *p* = 0.722, η_p_^2^ = 0.01).

### 3.2 Turning decisions

**Figure 4:**
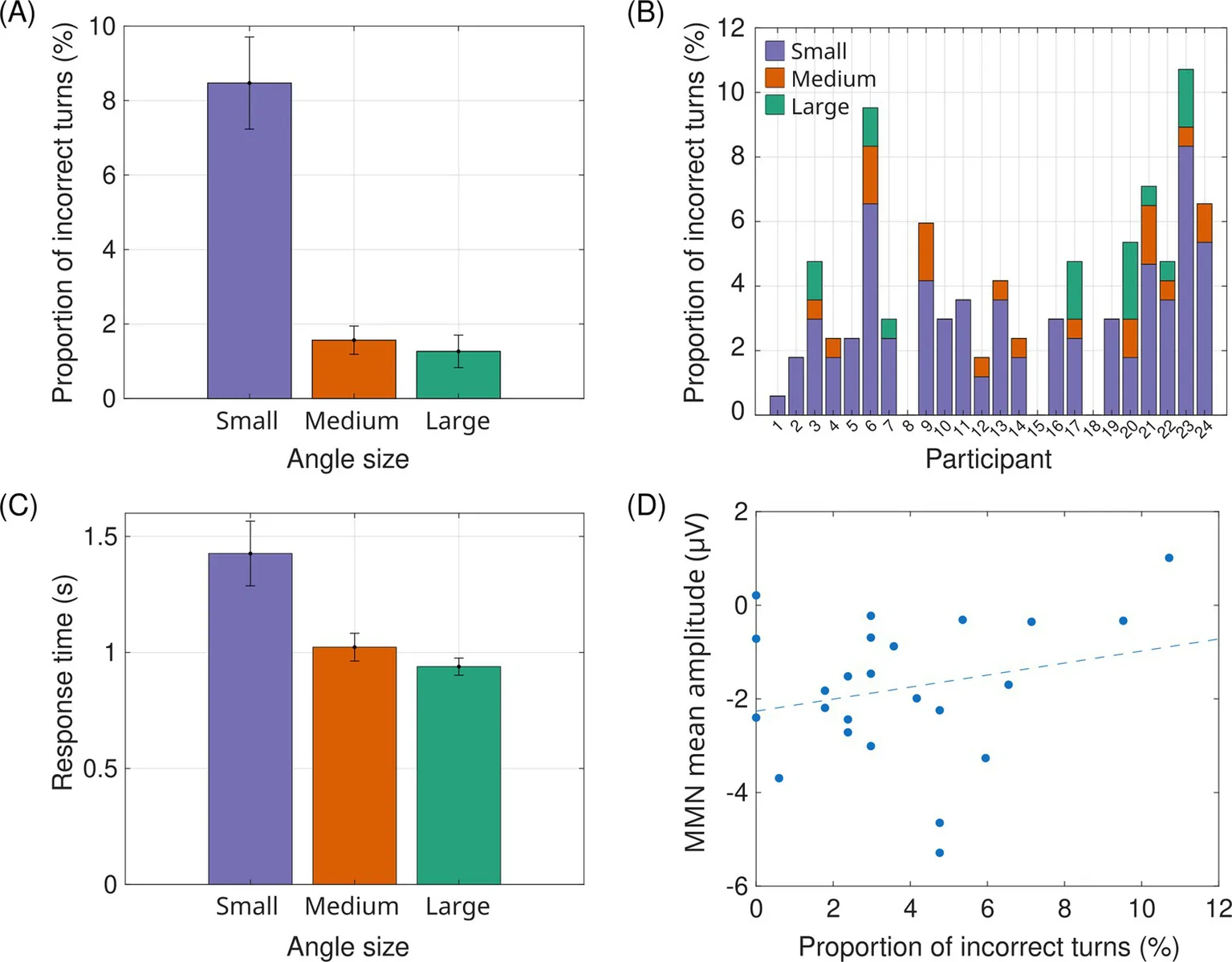
Turning behavior and its relationship with MMN amplitude. **(A)** Proportion of incorrect turning decisions for small, medium, and large angles between the participant’s walking direction and the alarm sound position. Error bars denote the standard error of the mean (SEM). **(B)** Proportion of incorrect turning decisions for each participant. Colored segments indicate the proportion of incorrect decisions that fell into the categories of small, medium, and large angles, respectively. **(C)** Mean response times for turning decisions in case of small, medium, and large angles between the participant’s walking direction and the alarm sound position. Error bars denote SEM. **(D)** Relationship between proportion of incorrect turns and MMN amplitude with regression line [note that the correlation is not significant, *r*(22) = 0.235, *p* = 0.269].

Each participant made 168 turning decisions in total. On average they turned in the wrong direction just 7.25 times (4.40% of the turning decisions) with a standard deviation of 4.30 (2.56%) and in a range from 0 to 18 (0 to 10.71%) wrong decisions (Figures 4A,B). This corresponds to the high homogeneity of paths taken through the maze (Figure 2B). Some participants reported that they sometimes accidentally clicked on the wrong side of the controller’s trackpad and therefore turned to the wrong direction without intention. This could explain why some wrong turns happened even with very large angles to the alarm, where the decision whether the sound source is on the left or the right side should be straightforward (Carlini et al., 2024). Overall though, as expected, errors occurred more often for smaller angles to the alarm source (8.47% wrong decisions for small angles, 1.57% for medium angles, 1.26% for large angles). The reliability of these differences was confirmed by a significant effect of angle size on correctness (*F*(2, 46) = 37.21, *p* < 0.001, η_p_^2^ = 0.62), with follow-up *t*-tests showing significant differences in correctness between small and medium angles (*t*(23) = 6.42, *p* < 0.001, *d* = 1.31) as well as between small and large angles (*t*(23) = 6.28, *p* < 0.001, *d* = 1.28), but not between medium and large angles (*t*(23) = 0.67, *p =* 0.512, *d* = 0.14).

Response times of the turning decisions (Figure 4C) were likewise modulated by angle size, (*F*(2, 46) = 14.73, *p* < 0.001, η_p_^2^ = 0.39), with more time taken for small angles (1.426 s) than for medium (1.023 s) and large angles (0.939 s). Follow-up *t*-tests showed significant response time differences between small and medium angles (*t*(23) = 3.86, *p* < 0.001, *d* = 0.79) as well as between small and large angles (*t*(23) = 3.95, *p* < 0.001, *d* = 0.81), but not between medium and large angles (*t*(23) = 2.15, *p =* 0.042, *d* = 0.44) against a Bonferroni-corrected alpha level of 0.0167.

The correlation of individual MMN amplitude at FCz and the proportion of errors (Figure 4D) was numerically positive as expected, but not significant, *r*(22) = 0.235, *p* = 0.269.

### 3.3 Head movements

**Figure 5:**
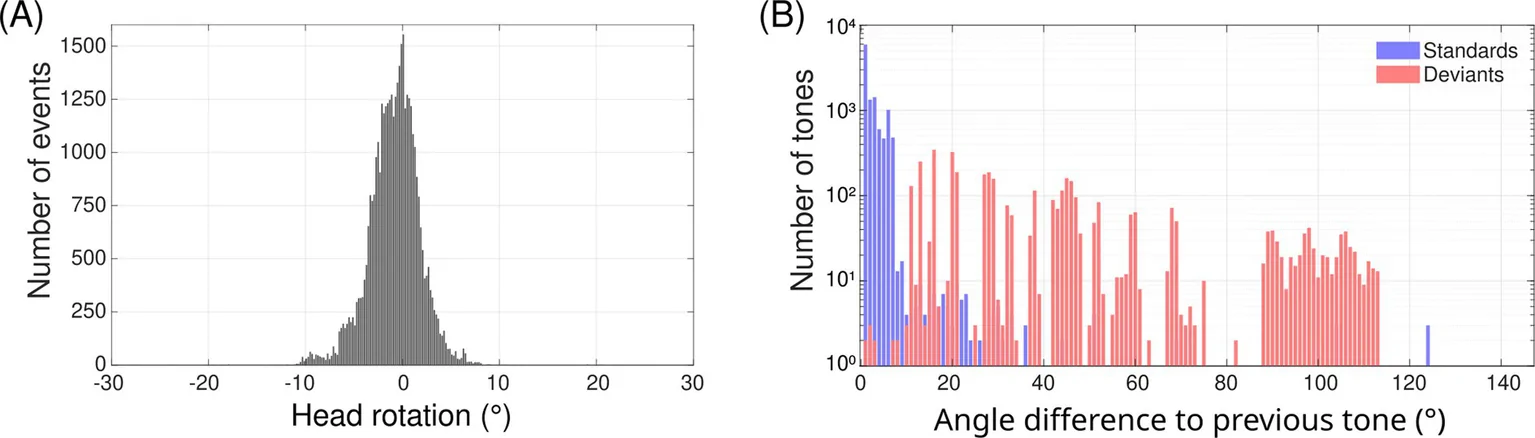
**(A)** Distribution of azimuthal head orientation at the time points of sound onset in oddball areas, binned into 0.2°-intervals and pooled across participants. Negative values indicate orientation to the left. **(B)** Distribution of angle differences between the current and the previous tone for standards (blue) and deviants (red). For better visibility, the *Y* axis is plotted on a logarithmic scale.

The relative distance and angle between participant and alarm changed as the participants moved through the maze (Figures 2C–E). In addition to (simulated) movement of the whole body towards the sound source, (real) head movements would alter the angle to the sound source more extensively and on a shorter time scale. To investigate the amount of head movements that actually happened during the experiment, we analyzed lateral head orientation during tone onsets while participants moved through the oddball areas (Figure 5A). It turns out that participants moved their heads very little, as 99.28% of these data points lay between −10° and 10°, with 0° meaning that the participants looked straight ahead in direction of the corridor, and positive values meaning the participants moved their head to the right. Mean head orientation during tone onsets in the oddball areas was −0.86° with a median of −0.70° and a standard deviation of 3.01°. Numerically, there is a slight trend towards head orientation to the left, but that trend is not significant (*t*(23) = −2.02, *p* = 0.055, *d* = −0.41).

Since participants did not move their heads very much while moving through the oddball areas, the sound-source angles (relative to the current head orientation) differed only little from one standard alarm tone to the next (average difference: 1.35°, standard deviation: 2.87°). This standard-to-standard difference was calculated across all oddball-area standards of all participants, excluding the standards right after a deviant. The variance is dominated by the effect of participants’ locomotion (which affect the sound source angles more strongly for corridors closer to the alarm) rather than by the effect of individual head movements. The difference between the angles to the alarm of the first and last standard stimulus in each oddball area was on average 9.40° (with a standard deviation of 13.78°, again dominated by angle differences between corridors). The angle to the alarm for deviants, on the other hand, differed a lot more from the preceding standard stimulus, with 42.51° on average and a standard deviation of 28.53° (Figure 4B).

## 4 Discussion

In this study, participants moved through a virtual maze while searching for the source of a repeating alarm tone. The acoustic scene was realistically simulated with TASCAR (Grimm et al., 2015, 2018, 2019), such that the acoustic properties of the alarm tone (distance, level, angle) naturally changed with participants’ body and head movements. We measured participants’ ability to localize the sound source by the correctness of their behavioral turning decisions in the maze, and by neural (EEG) correlates of detecting occasional, task-unrelated lateral displacements of the alarm tone.

The EEG data show the elicitation of a robust MMN component by laterally displaced (deviant) tones in the VR. MMN amplitude was significantly modulated by angle size, though the pair-wise follow-up comparisons failed to reach significance with conservative Bonferroni correction. This might partly be an issue of signal-to-noise ratio: An average of only 54 deviants per participant and angle size is much lower than typical recommendations for MMN recordings (Duncan et al., 2009). Higher MMN amplitude for large than for small spatial deviations, as descriptively observed here, is consistent with earlier studies (Paavilainen et al., 1989; Deouell et al., 2006). Note that the “small” deviations in our study (5.47° to 22.64°) by far exceed the minimum audible angle of 1° (Mills, 1958; Perrott and Saberi, 1990)—but this angle is observed in participants who actively try to find spatial differences, whereas our participants’ task instruction was not related to the deviant tones. The early peak latency (128 ms after stimulus onset) and the topography with a frontocentral maximum and polarity inversion at the mastoid electrodes indicates that the MMN was not overlaid by an N2b component (a negativity with central maximum and without polarity inversion, which occurs at around 200 ms after stimulus onset; Novak et al., 1990). This indicates that deviance detection happened on an auditory-sensory level, rather than within a process of explicit target detection.

The fact that the MMN was elicited by spatial deviants although there was no physically constant standard, confirms our hypothesis: The auditory system responds to deviations on a sensory level even when the spatial properties of consecutive standards are also changing due to the listener’s own movement. Our findings are consistent with earlier studies that observed an MMN in response to spatial deviants during head movements (Schechtman et al., 2012). Unlike these earlier studies, we used sine tones rather than noise bursts because we wanted to create the realistic impression of an alarm sound. Although sine tones are typically more difficult to localize than noise bursts (Middlebrooks and Green, 1991; Blauert, 1997), our results still show a clear MMN, encouraging us to use this contextually meaningful stimulus to further specify the observed effects and their implications for auditory scene analysis and mechanisms of auditory location processing.

As the spatial changes between consecutive standard tones were caused by the participant’s own movement, it is tempting to conclude that such movements were incorporated into the predictive model of the expected sound source’s angle and distance (Winkler et al., 1996; Garrido et al., 2009). An auditory predictive model that comprises the own movement’s consequences would be able to tell apart changes brought about by own movement from changes in the sound source’s location. This, in turn, would lead to MMN elicitation in our design. Our results are thus compatible with a predictive-coding-based explanation. However, they are also compatible with alternative interpretations: We have to acknowledge that the actual angular differences between deviants and standards were considerably higher than those between consecutive standards. Therefore, we cannot rule out the possibility that our deviance-related response was elicited due to this physical dissimilarity (Winkler et al., 1990), rather than because the MMN-generating system took head and body movements into account for predicting the next tone’s spatial properties. Physical dissimilarity would lead to differential adaptation for deviant and standard sounds, which would fully explain MMN generation without predictive modeling (May and Tiitinen, 2010; May, 2021). Beyond the theoretical controversy regarding the mechanisms underlying MMN elicitation (Näätänen et al., 2005; Fishman, 2014; Malmierca et al., 2014), this would open up interpretations of the present finding without recurrence to internal models, such as expressed in ecological resonance and embodied cognition approaches (e.g., Raja and Gramann, 2025).

One obvious way to counteract the physical dissimilarity between deviant and standard stimuli would be to increase the angular difference between consecutive standards. This would allow for a stricter test of the underlying predictive-model assumption. Yet the amount of spatial change caused by forward movement of the whole body is limited by the size of the VR and a reasonable choice of VR movement speed that does not lead to simulator sickness. For enhancing the amount of movements that would be performed (and the real-world-likeness of the experiment), participants were allowed to move their heads freely, which they could have used to gather additional localization cues (Carlile and Leung, 2016; Tsuji et al., 2024; Riedel et al., 2025). However, while walking through the maze corridors, participants actually did not move their heads a lot. In future studies with this setup, this could be overcome by adding tasks or changing the visual context in a way that would encourage participants to move their heads more extensively (as long as EEG artifacts remain within limits).

The suggestion to exert experimental control over the amount of participants’ head movements may sound counterintuitive from the perspective of naturalistic behavior. Yet the purpose of the approach developed in this study is not to examine participants’ behavior with as many degrees of freedom as possible—as pursued, for instance, in studying spatial navigation in less constrained settings following the framework of ecological psychology (Gramann et al., 2014; Stangl et al., 2023). Rather, our purpose here is to find a middle ground between experimental control and contextual plausibility for the participant. Simply instructing participants to move their heads more would come at the expense of artificiality, whereas giving them a reason to move their heads within the experimental context (e.g., because there is something interesting to see at the walls of the maze) keeps the natural character of the overall scene and setting.

With extensions along these lines, the framework developed in this study could prove useful for further investigations of dynamic sound source localization that would allow for stronger mechanistic conclusions. The difference of our approach to many earlier studies (Paavilainen et al., 1989; Deouell et al., 2006; Altmann et al., 2009; Schechtman et al., 2012) is that we chose a setup that feels more realistic to participants. This pertains to the VR itself, which simulates actual movement in space, but also to the embedding of the oddball sequence in an alarm search task. Though popular, the oddball paradigm often suffers from being artificially added to a lab or VR scene, while here we created an authentic reason (the alarm) for the repetitive tones to be played. Similar to our recent work that puts the self-generation of tones into a plausible scenery (Feder et al., 2023), this enhanced the motivational character of the experiment for participants. While alarm sources could of course be searched in a real environment as well, the space demands would be prohibitive (the size of the virtual maze corresponded to 115.5 m by 52.5 m in real space). Using VR also allows to create an authentic representation of the real world without risking to lose control over the experimental setup (Stodt et al., 2025). For instance, we were able to ad-hoc modify the maze when participants took unexpected turns, without them noticing that we had done so. This enabled us to guide the participants’ movements in an experimentally relevant range without explicit error feedback or noticeable setting manipulations that would violate naturalness.

In some respects, the naturalness of the paradigm is yet to be improved in future studies. The fact that participants were sitting rather than standing, and moving forward automatically rather than actually moving in space, deprived them of an important part of the motor-sensory feedback. While the space demands for real walking were prohibitive, a compromise might be simulated forward walking by combining VR with a treadmill. Conventional VR treadmills make this feasible for straight-ahead locomotion, yet our maze design would require an omnidirectional treadmill to allow for implementing turning decisions as well. The turns themselves present another limitation to the naturalness of the design: Turning decisions were made by clicking on the controller’s trackpad, and they only changed the virtual orientation but not the actual orientation of the participant in space. Again, this may have impoverished the motor-sensory feedback. Alternatives would be to automatically rotate the experimental chair in real space on the basis of the participant’s decision, or to have participants stand (walk-in-place) and indicate their turning decisions by body rotation (Drewes et al., 2021; Feder et al., 2022). Finally, the teleportation element may have further diminished naturalness, but since this only happened at the end of the walkthrough and only in the rare cases where participants did not arrive at the alarm (6 times out of 576 walkthroughs summed across participants), a strong effect of this is unlikely.

The actual task of finding the alarm source seemed to be rather easy for participants: wrong turning decisions happened only rarely. This is not too surprising for turning points with large lateral angles, where the decision whether the alarm origin is on the left or right side is easy (Carlini et al., 2024). Smaller lateral angles to the alarm source increased the number of wrong turning decisions; nevertheless, performance remained astonishingly good throughout. Ceiling effects may have diminished the observable size of the correlation between EEG and behavioral measures of sound localization, though this remains to be determined in future studies with higher localization difficulty. Some participants reported using the deviants to find out on which side the alarm is positioned. Even though they were never explicitly told, they realized that the alarm always switched sides for one tone relative to the midline, and that this information could help them to conclude that the standard tone must be on the other side. This strategy was not taken into account in the planning of the study, and should be avoided in future studies by presenting deviants to either side of the standard. Nevertheless, it is unlikely that the strategy was used abundantly, as this would be visible by N2b contributions to the MMN component, which—as discussed above—were not observed.

The experimental setup developed here and the results obtained so far provide a promising framework and proof-of-concept for follow-up studies on sound localization under dynamic listening requirements. For successful auditory scene analysis, it is certainly important to tell apart spatial changes caused by own movement and by actual movement of the sound source. The present work opens up further possibilities for investigating the mechanisms that allow us to cause and interpret sensory changes at the same time, and the possible role of predictive mental models therein.

## Data Availability

The pre-processed EEG data (single-subject averages) and behavioral data supporting the conclusions of this article are available alongside with the analysis code in a publicly accessible repository (https://osf.io/ymgw3/). The raw EEG data will be made available by the authors upon request, without undue reservation.

## Footnotes

1 Note that the author Sabine Grimm of the present paper is unrelated to Giso Grimm, the core developer of the TASCAR framework.

2
https://jackaudio.org/

3
http://uniosc.monoflow.org/
